# Formation of SiC nanoparticles in an atmospheric microwave plasma

**DOI:** 10.3762/bjnano.2.71

**Published:** 2011-10-07

**Authors:** Martin Vennekamp, Ingolf Bauer, Matthias Groh, Evgeni Sperling, Susanne Ueberlein, Maksym Myndyk, Gerrit Mäder, Stefan Kaskel

**Affiliations:** 1 TU Dresden, Faculty of Science, Department of Chemistry and Food Chemistry, 01062 Dresden, Germany; 2 Fraunhofer Institute Materials and Beam Technology, Winterbergstrasse 28, 01277 Dresden, Germany

**Keywords:** atmospheric microwave plasma, nanoparticle, SiC

## Abstract

We describe the formation of SiC nanopowder using an atmospheric argon microwave plasma with tetramethylsilane (TMS) as precursor. The impact of several process conditions on the particle size of the product is experimentally investigated. Particles with sizes ranging from 7 nm to about 20 nm according to BET and XRD measurements are produced. The dependency of the particle size on the process parameters is evaluated statistically and explained with growth-rate equations derived from the theory of Ostwald ripening. The results show that the particle size is mainly influenced by the concentration of the precursor material in the plasma.

## Introduction

Silicon Carbide (SiC) is a solid with various applications in materials science. It is used, e.g., as a wear-resistant material, as a heterogeneous catalyst, and in the production of semiconductors. There, SiC layers are deposited as low-k copper diffusion barriers by the application of organic precursors in plasma processes [1–2], and preventing the formation of SiC nanoparticles as a defect source is a challenge in this established industrial process. But, SiC nanoparticles also exhibit properties different from the bulk material and allow the creation of composite materials with new properties. Thus, their production has been studied by different methods such as the thermal pyrolysis of organic precursors [3–5], or plasma synthesis by means of DC thermal [6], inductive [7–8] or low-pressure microwave plasmas [9].

Even though the plasma synthesis of several materials has been investigated [10–17], only a limited number of experiments have been performed on the synthesis of nanoparticles in an atmospheric plasma [18–19]. However, the established industrial production processes for nanoparticles, such as flame pyrolysis yielding millions of tons of carbon black or oxide nanoparticles per year, are performed at atmospheric pressure. The plasma synthesis of nanoparticles in atmospheric plasma offers the advantage of a smaller experimental effort and thus lower costs, opening up a route to new applications of the materials. We propose a two-step approach for the design of a synthesis route: Firstly, one needs a basic theoretical understanding of the processes that lead to the formation of the desired powders. This allows one to find a promising starting point for the fine adjustment of the synthesis. Secondly, a systematic investigation of the impact of the system parameters on the product properties is required in order to find an optimized process point for a stable production. Due to its high mechanical toughness at evaluated temperatures, and the extreme hardness and chemical resistance of SiC, it is often used as welding flux. Phase separation during the welding process is a major technical challenge, and using nanosized particles may help to overcome this problem. So far, no synthesis of SiC nanoparticles in an atmospheric plasma has been published. We present a production method using an atmospheric plasma torch in a plasma reactor that was built for this investigation. The SiC synthesis was used as a model experiment to analyse the impact of the process parameter on the particle size in this approach.

## Experimental

The atmospheric plasma equipment consists of a 2000 W commercial microwave source (MX2000D), 3-stub-Tuner and atmospheric plasma applicator (MP-APS-03) from Muegge Electronics GmbH (Germany). The applicator head holds a quartz glass tube (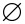
_outer_ = 30 mm; 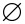
_inner_ = 27 mm; length = 1000 mm), which is open to atmosphere, but equipped with a suction system for the exhaust. The applicator has two tangential gas inlets for a swirl gas (“sheath”) and a central gas inlet for the reaction mixture (“core”); this setup realises a vortex-type mixing of the gases. The gas flow is controlled by an MFC (Ar swirl gas: 250 L_n_/min; H_2_: 500 mL_n_/min) while the feed of the liquid precursor is controlled by a LFC (TMS: 25 g/h). The liquid is taken from a pressurised reservoir (WaVo), then evaporated and mixed with Ar (500 mL_n_/min) in a Controlled Evaporation Mixer (CEM; all gas line parts from Bronkhorst High-Tech B.V.). The whole system is connected to a computer and is controlled by an in-house-developed LABVIEW program.

In the atmospheric pressure reactor an argon plasma ignites at gas flows between 20 to 180 L_n_/min with microwave powers above 1100 W. A stable discharge can be sustained down to a microwave power of approximately 200 W, with lower gas flows resulting in a more stable plasma state. Depending on the gas flow and the microwave power the plasma burns either in the swirl gas zone (low gas flow and high power, e.g., at 1500 W and 50 L_n_/min) or in the centre of the reactor tube (high gas flow and low power, e.g., at 700 W and 150 L_n_/min).

Data from the equipment were logged electronically and used to calculate the means and standard deviations of the gas flows and the microwave power. During each individual experiment the gas flows were run first, then the microwave power was engaged and the plasma was ignited. The reflected power was tuned to a minimum value by arranging the 3-stub tuner. The nanoparticles accumulated in the apparatus and were collected mechanically after the experiment by means of customised PTFE tools.

The surface area of the produced powder was measured with the BET (Brunauer, Emmett, and Teller) method in a QUADRASORB Sl (Quantachrome Instruments). The specific surface area σ (m^2^/g) gives the particle diameter *d* (nm) as *d*_BET_ = 6000/(σ∙ρ), when we assume spherical and monodisperse particles with bulk density ρ (g/cm^3^). Additionally, X-ray diffractograms were measured on a STOE StadiP instrument with an image plate detector. From the line broadening of the reflection peaks the particle size can be calculated from the Scherrer equation under the assumption of a crystalline structure of the powder. For this purpose the software WinXPOW (STOE & Cie GmbH) and LaB_6_ as line-width standard was employed.

The dependence of the product properties on the machine parameters was investigated by means of a particular experimental design [20]. The parameters were varied in a systematic way within the margins of the possible range, see Table 1. Within the experiments several parameters were changed simultaneously. The design spans the maximum experimental parameter space and was computed with the aid of a commercial software program (SAS jmp). The measured properties of the product are regarded as a supposed linear response on these parameters. The microwave power was varied in total by a factor of 2.8, the Ar flow by a factor of 12.5, the H_2_ and Ar (core) flow by a factor of 9.0 and the TMS flow by a factor of 6.7. It can be argued that this range exceeds the linear regime of the response functions, but nevertheless an overview of the impact of the newly built system parameters was obtained by the applied method in an efficient way, which justifies the chosen screening method. Additionally, three centre point experiments were performed using the mean settings, in order to prove the repeatability of experiments. A summary of the value combinations used in the performed experiments can be found below in Table 2.

**Table 1:** Summary of the experimental set points for the experimental design (design of experiment, DoE).

parameter	unit	centre point	low	high

microwave power	[W]	1250	650	1800
Ar (sheath)	[L_n_/min]	55	10	125
H_2_ (core)	[mL_n_/min]	250	50	450
Ar (core)	[mL_n_/min]	300	50	450
TMS (core)	[g/h]	5.75	1.5	10

## Results

The generation of charged gaseous species, which mark the plasma state, is usually performed by applying an electric field to a gas. Initially, ionised particles and free electrons in the gas (generated by natural sources or by a high voltage pulse to the system) will be accelerated in an electric field and ionise additional atoms or molecules by collision ionisation, creating a cascade of ionised species, which sustains the plasma. Ions in the plasma will be transported by ambipolar diffusion, which makes the electron temperature the most important factor for the ion diffusion in the plasma [21]. In turn the transport coefficient of these particles determines the growth kinetics of the nanoparticles. The majority of ions and hot electrons in our experiments were produced from the excited argon sheath gas.

By reacting the TMS precursor in the vapour phase with plasma-induced hot electrons, the rate-limited nucleation reaction step is accelerated and a significantly higher reaction rate is sustained at lower temperatures than is possible with thermal activation alone [9]. An important advantage of the plasma process for nanoparticle synthesis is that the electrons escaping from the plasma zone are attached to surfaces in contact with the plasma, causing a negative charge at the plasma boundary and a positive bulk plasma potential. A negative charge on the nanoparticle surface suppresses their agglomeration [9,21], making the plasma synthesis an ideal tool for nanoparticle formation.

In a gaseous plasma there exist several radical and ion species, which are formed by the decomposition of the feed gases [9,22]. The system will minimize its internal energy, if possible, by building molecules or clusters. Small particles of SiC as a thermodynamically stable compound will be formed in this experiment. In a first-order approximation the particles are spherical, and the vapour pressure of a curved surface *p*_v_ is higher than that of a flat surface *p*_∞_. This is expressed by the Gibbs–Thompson law *p*_v_ = *p*_∞_·exp(2σ*v*_m_/(*rRT*)), with σ as the free surface energy, *v*_m_ as the molar volume, *R* as the gas constant and *T* as the absolute temperature. Solving the equilibrium equation for the excess surface energy of a small particle on the one hand, and the heat of condensation on the other hand, gives an expression for the minimum diameter of stable particles. The critical diameter *r*_crit_ is given by

[1]
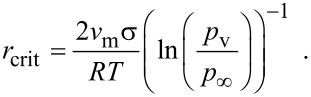


The minimum stable particle diameter increases directly with the reciprocal temperature 1/*T* and with the reciprocal difference between the logarithms of the vapour pressure of the decomposed precursor and that of the product 1/(ln *p*_v_ − ln *p*_∞_).

To give an estimation of the critical diameter, the values in Equation 1 can be approximated as follows: The typical gas temperature for an atmospheric gas plasma is about 2000 K [23], the vapour pressure of SiC at this temperature is in the order of *p*_∞_ ≈ 0.2 Pa [24], the surface energy of SiC is σ ≈ 2000 mN/m [25], and the molar volume is usually assumed to be that of the bulk material.

**Figure 1:**
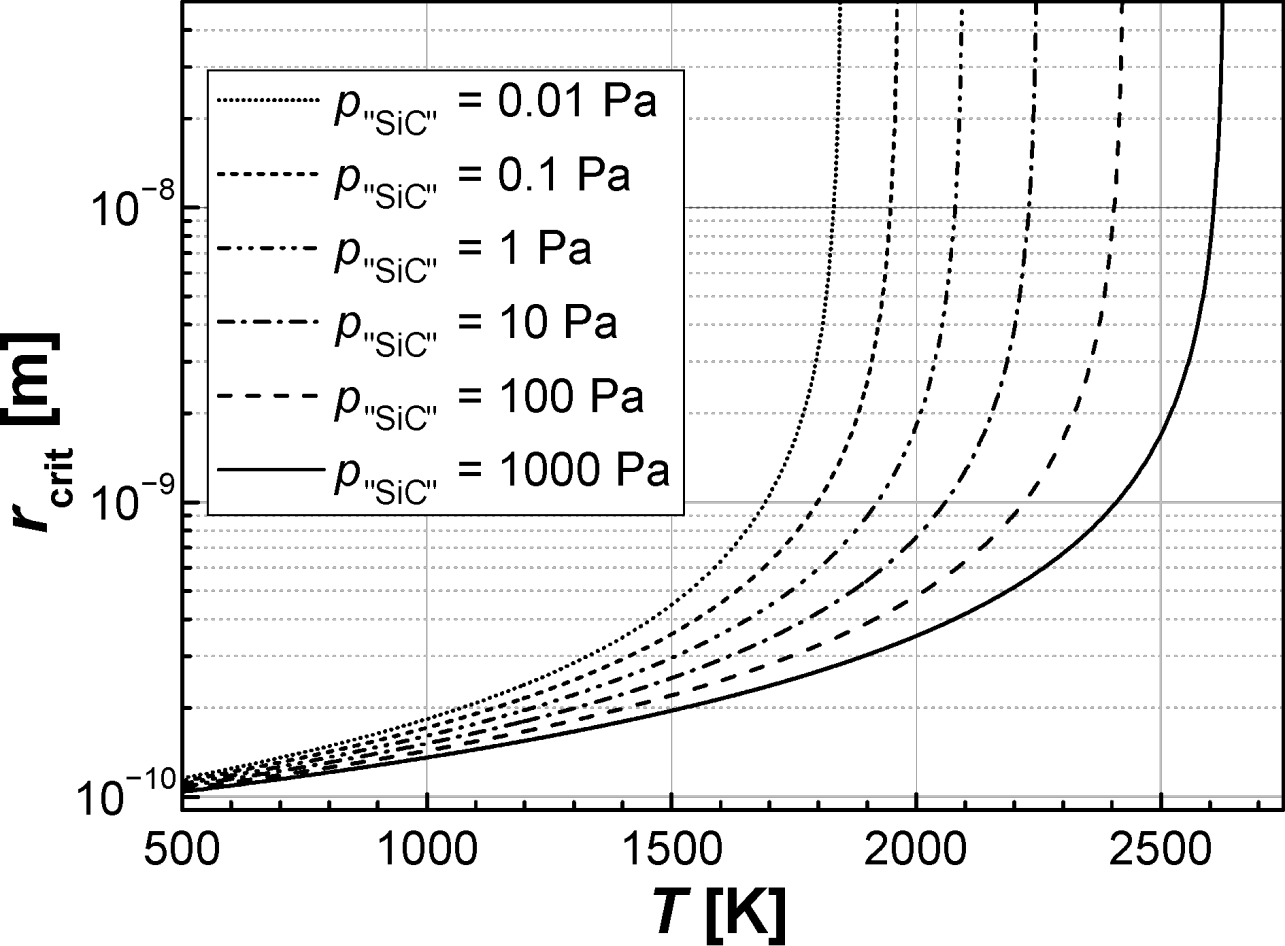
Calculated values for the smallest thermodynamically stable particle radius *r*_crit_ at different partial pressures of gaseous "SiC" species, according to Equation 1.

Hence, in order to determine the minimal nanoparticle size during the microwave synthesis we made the following arbitrary assumption: The partial pressure of the SiC species in the gas phase over the SiC nanoparticles is determined by the precursor concentration, assuming a complete decomposition of the precursor in the atmospheric plasma into building units for the nanoparticles. Typical precursor values are in the range of 1000 ppm, which results in a gas pressure of *p*_v_ ≈ *p*_"SiC"_ ≈ 100 Pa. This leads to a value for the minimum nanoparticle diameter in the order of half a nanometer (Figure 1).

In order to attach additional material to the initially formed particles, material has to be transported to its surface and incorporated into the particle. A formal description of this reaction is usually referred to as the Lifshitz–Slyozov–Wagner (LSW) theory [26–27], which has also been adopted for description of the growth of nanoparticles from the gas phase [28].

The surface kinetics in a plasma is increased [29], driving the growth process into a diffusion limit. The particle growth rate is therefore given by:

[2]
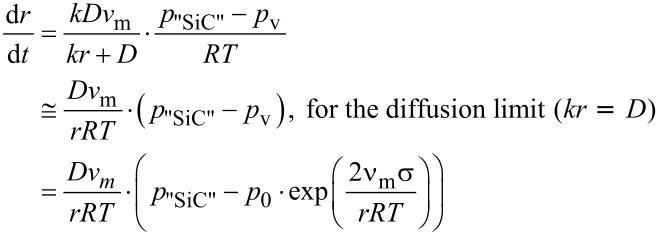


where *k* is the rate of the surface reaction with the nanoparticle, *D* the diffusion coefficient of the educt species in the (atmospheric) gaseous plasma, *p*_v_ is given by the Gibbs–Thompson law, and again *p*_"SiC"_ can be approximated based on the concentration of the precursor for the nanoparticle. The equation describes the growth rate of larger particles, which is known as Ostwald ripening, resulting in the well-known log-normal distribution of the particle sizes.

**Figure 2:**
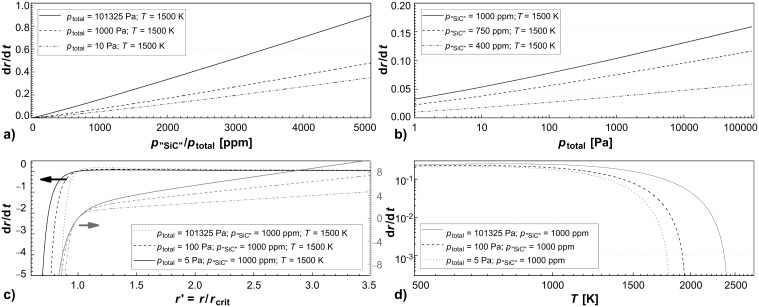
Visualisation of the relation between the growth d*r/*d*t* and the different variables from Equation 2. The growth rate d*r*/d*t* a) depends linearly on the concentration of the precursor species *c*_precursor_ ≡ *p*_"SiC"_/*p*_total_, b) is a linear function of the logarithm of the pressure, when the concentration of the precursor species is kept constant, c) increases linearly with the particle volume *V* for particle sizes above the critical radius *r*_crit_ (cf. Equation 1), and d) shows only a small dependency for low temperatures but decays rapidly for higher temperatures.

Taking into account, that *D* = ƒ(1 = *p*_total_, *T*^x^), with 1 ≤ x ≤ 2, one can give the following proportionalities for the growth rate of a nanoparticle for a large supersaturation of the system *p*_"SiC"_ >> *p*_v_, as depicted in Figure 2:

[3]
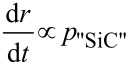


[4]
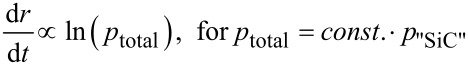


[5]
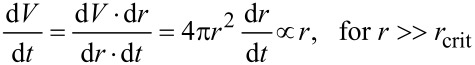


The high ambipolar diffusion coefficient of the species in the plasma results in the fast growth of the particle accompanied by the fast depletion of the educt in the plasma, which of course contradicts the simple assumption of a constant *p*_"SiC"_ as made for the figures. Nevertheless, we think that the system achieves a steady state due to the steady addition of the feed gas and constant microwave energy, making the derived proportionalities a valid snapshot of the growth kinetics at least in a spatially limited part of the reactor. It can also be argued that the particle growth rate is a function of the investigated factors d*r* = d*t* = ƒ(*p*_"SiC"_, *p*_total_, *T*, *r*,…) and that the factors in Equation 3, Equation 4, and Equation 5 present the first derivatives in a Taylor series.

The results of the experiments are shown in Table 2. SiC particles in the size range of 7 nm to 22 nm were synthesised. Usually the product was formed as a black powder, except for in the case of experiment II and experiment IV (light brown/brown powder). All samples were investigated with XRD and adsorption measurements (*BET*) to calculate the particle sizes. Some of the samples were additionally investigated with TEM measurements to verify the data. Infrared spectroscopy in combination with annealing experiments was performed to gain an insight into the composition of the samples.

**Table 2:** Summary of the experimental conditions (as a mean of the electronically logged experimental data) and the obtained results for the particle diameter from the BET and the XRD measurement.

exp. nr.	microwave power (fwd.) [W]	Ar (sheath) [L_n_/min]	H_2_ (core) [mL_n_/min]	Ar (core) [mL_n_/min]	TMS (core) [g/h]	duration [min]	*d*_BET_ [nm]	*d*_XRD_ [nm]

Ia	1137	55	250	300	5.72	30.8	7.62	8.02
Ib	1101	55	250	300	5.73	30.1	8.12	9.50
Ic	1137	55	250	300	5.72	30.2	7.91	7.32
II	1786	121	50	50	1.44	130.1	8.83	8.36
III	637	10	450	50	10.00	29.9	16.14	11.98
IV	1799	125	450	50	10.03	30.5	6.96	9.60
V	1601	10	50	50	1.49	59.6	9.77	10.92
VI	637	10	50	50	1.50	119.9	8.80	15.31
VII	1797	10	50	450	10.03	30.5	15.84	21.69
VIII	1702	10	450	450	1.48	33.2	11.21	7.98

*BET and XRD:* The X-ray measurements of the samples show typical reflections for β-SiC. The diffraction peaks due to the nanosize of the particle are broad, which allows the calculation of the particle size using the Scherrer equation. For most experiments, the particle sizes calculated from XRD and adsorption measurements are in good agreement. Higher deviations between the two applied measurement methods are found for experiment IV, VI and VII (Table 2), showing significantly smaller particle sizes for BET compared to those obtained from XRD, and experiments III and VIII, with opposite deviation. Smaller particle sizes for XRD measurements can be explained by an amorphous phase in the samples and the agglomeration of the grains, which leads to a smaller effective surface area. The opposite deviation is sometimes explained by the presence of large particles in the samples, and indeed in the samples a few large particles were found. In some experiments, compact product layers were deposited in the area of the microwave applicator head. A contamination of the samples with particles from these layers could not be avoided when harvesting the product from the reactor.

**Figure 3:**
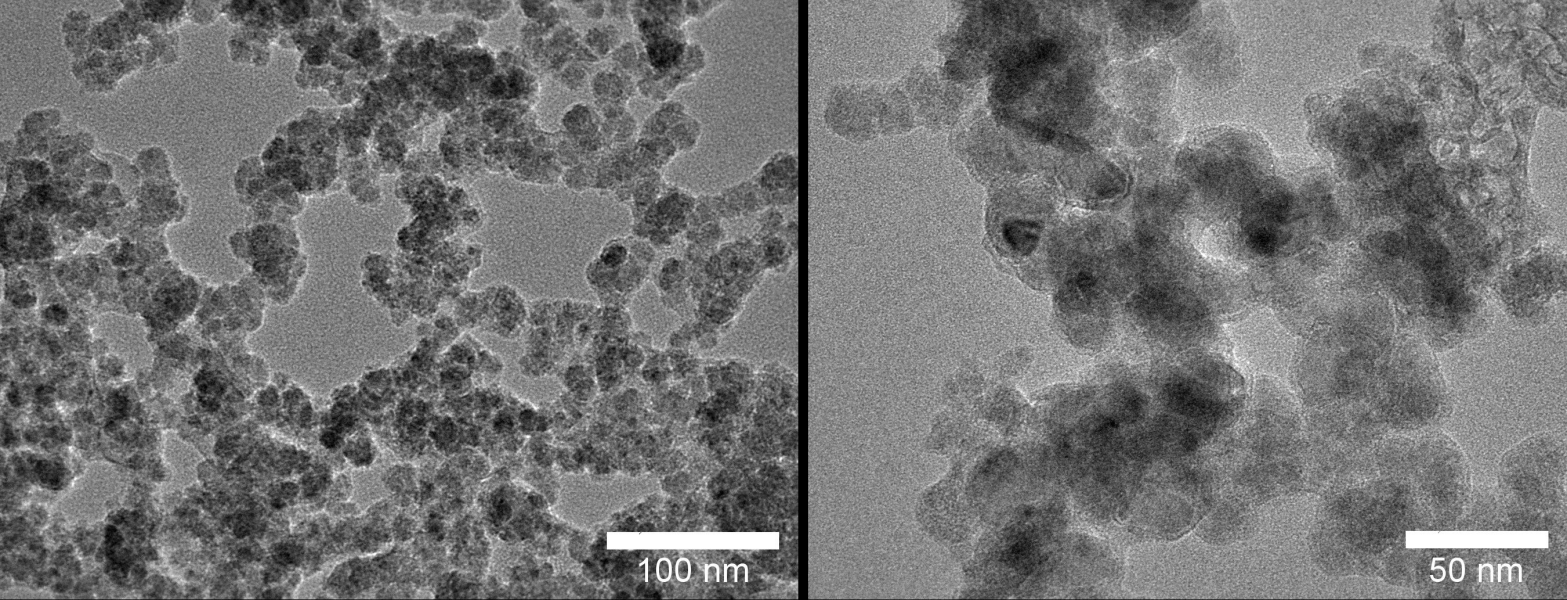
TEM images of the nanosized SiC samples synthesized in the atmospheric pressure microwave plasma reactor; the samples were taken from experiment III in Table 2.

*Transmission electron microscopy*: Electron micrographs of sample III are shown in Figure 3. Average particle size is about 10 nm, which is in good agreement with XRD result. Furthermore all particles are found to be agglomerated, which easily explains the higher particle size measured by the BET method. The agglomerated particles obey a smaller surface area, which calculates to larger average particle size.

**Figure 4:**
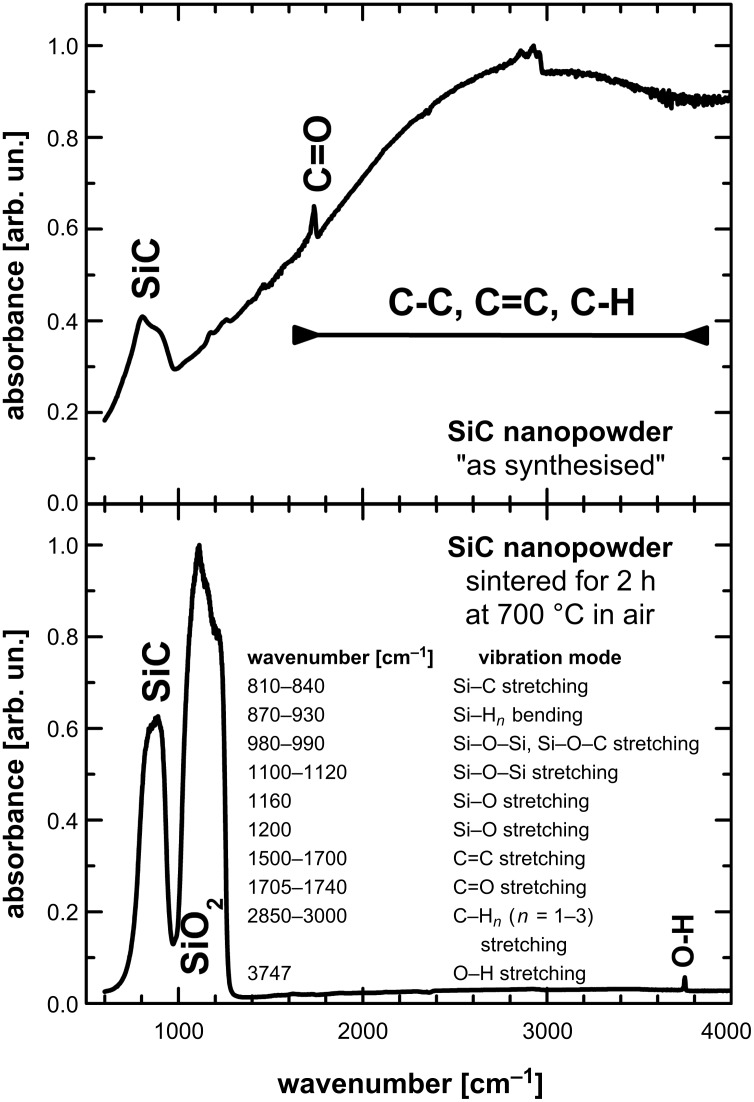
Infrared spectra of the sample number VII in Table 2, as synthesized and after calcinations in air at 700 °C. The thermal treatment changes the material colour from black to light grey. In the IR measurement carbon related signals disappear, while SiO_2_ modes show up.

*IR spectroscopy and thermal treatment:* The dark colour of the powders leads to the assumption that the particles are contaminated with excess carbon, at least on the surface of the particles. Thus we applied IR measurements as a surface sensitive method in addition to the XRD method. Typical results are shown in Figure 4; the as synthesized sample shows absorption modes that can be attributed to various carbon compounds [30–32]. McFarland et al. confirmed that carbon-rich SiC was formed in the argon plasma, even with addition of H_2_. By addition of hydrogen more H radicals are formed in the reactive plasma, and the excess of carbon can be reduced by the attack of highly reactive H radicals, and hence CH_4_ is released [9]. To remove the excess carbon in the sample, we also performed a heat treatment of the materials. SiC is stable in air at temperatures up to 700 °C, while free carbon is oxidized at temperatures between 530–680 °C [33]. After 2 hours at 700 °C the signals from the excess carbon disappeared, while SiO stretching modes showed up. During the heat treatment, the colour of the samples changed from dark to light grey. Applying temperatures above 700 °C or lower temperatures for several days reduced the intensity of the SiC peaks and an increased intensity of the SiO*_x_* signal was observed. The X-ray diffraction patterns of the untreated and annealed samples showed no significant difference, thus one can assume that glassy SiO_2_ was created.

**Figure 5:**
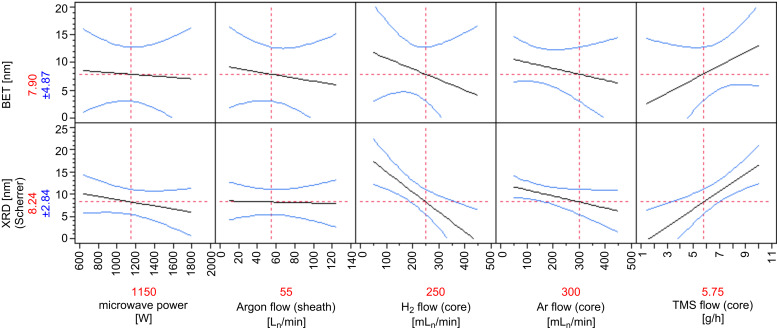
Response plot of the particle sizes based on adsorption measurements (BET, upper row) and on Scherrer’s equation (XRD, lower row). The black straight lines indicate the calculated linear dependence of the response value from the experimental factors. The blue curves show the 95% confidence limits of the linear correlations. The red dotted lines and values indicate the centre values of the factors. On the ordinate the calculated response values are shown with the error within the 95% confidence intervals in blue.

*Evaluation of the experimental design:* The designed experiments screen the response of the particle size to the equipment parameter settings. A statistical evaluation of the experiments leads to a system of linear equations describing the relation between the parameters and the responses. Fewer experiments lead to larger errors in the model, nevertheless the DoE method reveals general trends for the synthesis in the microwave system with a high level of confidence. The computed results are shown in Figure 5. Again, only small differences were found for the results based on the BET and XRD measurements, except for a varying impact of the hydrogen flow. It is straightforward to interpret this as a strong impact of the hydrogen on the crystallinity of the product. The other factors act as follows: The microwave power, argon core and argon sheath flow have a small negative impact. The educt flow shows a large positive effect on the nanoparticle size.

For the interpretation of the results we use the proportionality equations presented in the theory section of this article. The dependency of the particle growth rate on the precursor concentration was derived (Equation 3). This was confirmed by the huge effect of the TMS flow and the argon flow as sheath gas, leading to an increase or decrease of the precursor concentration. The argon flow in the core is much smaller, which corresponds to a smaller magnitude (see Table 1). Additionally, a higher flow rate causes a faster quenching of the reaction, simply due to the faster transport of the material out of the microwave-heated zone (Equation 5). In an opposite manner the microwave power increases the length of the reaction zone (i.e., the plasma torch) for the nanoparticle growth, which leads to longer residence time for the growth reaction. The addition of H_2_ into the argon plasma causes a higher plasma temperature, and also a higher neutral gas temperature and, secondly, helps to decrease the carbon content in the argon plasma.

Addition of hydrogen into the argon plasma will also increase the reaction rate owing to a higher diffusion coefficient of the species (Equation 2). On the other hand this experiment indicates a poorer crystallisation of the product, which is represented by a smaller effective particle size according to Equation 2.

## Discussion

The synthesis of SiC by means of an atmospheric microwave plasma as a source of thermal energy for the decomposition of TMS has been shown. The reaction is initiated by collisions between electrons and neutral molecules of TMS. By reacting the TMS precursors in the vapour phase with plasma-induced hot electrons, the nucleation reaction step is accelerated and a significantly higher reaction rate is sustained at lower temperatures than is possible with thermal activation alone [9].

The particle growth rate from the precursor concentration was derived as Equation 3 and it was confirmed by the effect of the TMS flow and the argon flow as sheath gas, leading to an increase or decrease of the precursor concentration. Additionally a higher flow rate of core argon gas causes a quenching of the reaction, due to the faster transport of the material out of the microwave-heated zone. In an opposite manner, the microwave power increases the length of the reaction zone, which leads to longer residence time for the growth reaction. The addition of hydrogen into the argon plasma causes an increase of H radicals, and the excess of carbon in SiC is reduced by methane formation.

Even though the plasma production of nanoparticles is an established industrial process, only a limited number of systematic investigations have been reported on this synthesis. Wiggers et al. investigated the formation of ZnO nanoparticles in a low-pressure microwave reactor by particle mass spectroscopy (PMS) [10]. In that work, about twenty *one-factor-at-a-time* experiments were evaluated, and a positive impact of the precursor concentration, a positive impact of the total pressure, and a negative impact of the microwave power on the mean particle size were reported. None of these relations were linear, and no parameter interactions were considered. The same authors reported the synthesis of silicon nanoparticles in a similar experimental setup [11]. In that experimental work, ten single experiments were presented and the qualitative correlations of the particle size with the process parameters were the same as in the investigation on ZnO. Nevertheless, in the case of the Si nanoparticle synthesis, an approximately five-times higher microwave power was applied, and compared to the previous work a different functional relation was observed, i.e., in the case of the previous low power synthesis the relation flattens towards the higher power limit, while it rises towards that limit in the high power case. Furthermore the silicon model system is theoretically described by a calculation based on the Fluent program [13]. The quantitative relations between precursor concentration, pressure and microwave power and the particle size were shown again. The experimental data were well reproduced by the calculations, but only two data points were created for each parameter in the experiment and in the respective calculations. The observed relations between the process parameters and the particle size are in good accordance to our own results. Furthermore, the increase of the particle size with the total pressure reflects a higher growth rate of the particles, as predicted by Equation 4.

The only published synthesis of nanosized SiC from tetramethylsilane (TMS) in a microwave plasma was reported by McFarland et al. [9]. In a low-pressure argon plasma (pressure between 1 to 10 mTorr) amorphous SiC nanoparticles with a size between 4 and 6 nm were synthesised; higher gas flows reduced the particle size. The overall smaller particle size of the nanoparticles in the previous work can be explained on the basis of our own findings by the lower pressure and the lower residence time of particles in the plasma.

## Conclusion

Silicon carbide nanoparticles were produced by means of atmospheric-microwave-plasma induced decomposition of tetramethylsilane. The reaction conditions were varied to produce nanoparticles with a diameter from 7 to 20 nm depending on the reaction conditions. The main parameter controlling the size of the nanoparticles is the concentration of the tetramethylsilane in the plasma, overruling an impact of the gas pressure significantly. The availability of modern plasma torches allows stable processing at atmospheric pressure, opening up a route for the large-scale production of nanoparticles with a relatively cheap instrumental setup. The general response of the product properties on the process parameters is no different from the response found in a low-pressure plasma. Using a set of designed experiments we were able to reduce the number of experiments needed to get a complete description of the experimental responses to the process parameters. Thus, the chosen "engineering approach" reduces the time and cost of the process development for the plasma synthesis. The newly developed tool will be used to investigate further materials, opening a route for the efficient creation of new applications of nanosized powders. The customised production of nanosized particles requires a theoretical understanding, but is also dependent on the instrumental details of the plasma equipment. We built an atmospheric plasma system for the creation of nanoparticles, which is capable of creating gaseous plasma suitable for that need, and the system was set up in a manner such that nanoparticles were synthesised in a cheap and reproducible manner.
